# Digital Ischemia as an Initial Presentation in a COVID-19-Positive Patient Without Any Significant Respiratory Symptoms

**DOI:** 10.7759/cureus.14054

**Published:** 2021-03-23

**Authors:** Trishna Acherjee, Barbara Bastien, Miguel A Rodriguez-Guerra, Syeda Salman, Nisha Ali

**Affiliations:** 1 Internal Medicine, BronxCare Health System, Bronx, USA

**Keywords:** digital ischemia, covid-19, lmwh, thrombosis

## Abstract

Coronavirus disease 2019 (COVID-19) is an evolving situation worldwide, which is associated with a broad range of symptoms from pneumonia/acute respiratory distress syndrome (ARDS) to multiorgan failure. So far, we have also encountered several patients with coagulopathy, including pulmonary embolism and deep vein thrombosis. A few cases of limb ischemia related to COVID-19 have been reported as well, but most of them involve critically ill patients. In this report, we discuss a case of COVID-19 in a patient who presented with right thumb ischemia without any significant respiratory symptoms.

## Introduction

In the early months of 2020, the USA's healthcare system was hit by a new pandemic caused by a novel coronavirus: severe acute respiratory syndrome coronavirus 2 (SARS-CoV-2), the causative organism behind the coronavirus disease 2019 (COVID-19). SARS-CoV-2 enters into the cells by binding with angiotensin-converting enzyme 2 (ACE2) receptor; since ACE2 is expressed in the endothelium, it may induce endothelial shedding and dysfunction, thereby contributing to vascular damage, local inflammation, and production of procoagulant factors predisposing individuals to thrombosis, similar to the increase in myocardial infarctions observed after influenza infections [1]. We report a case of digital ischemia as an atypical initial presentation in a patient diagnosed with COVID-19.

## Case presentation

**Figure 1:**
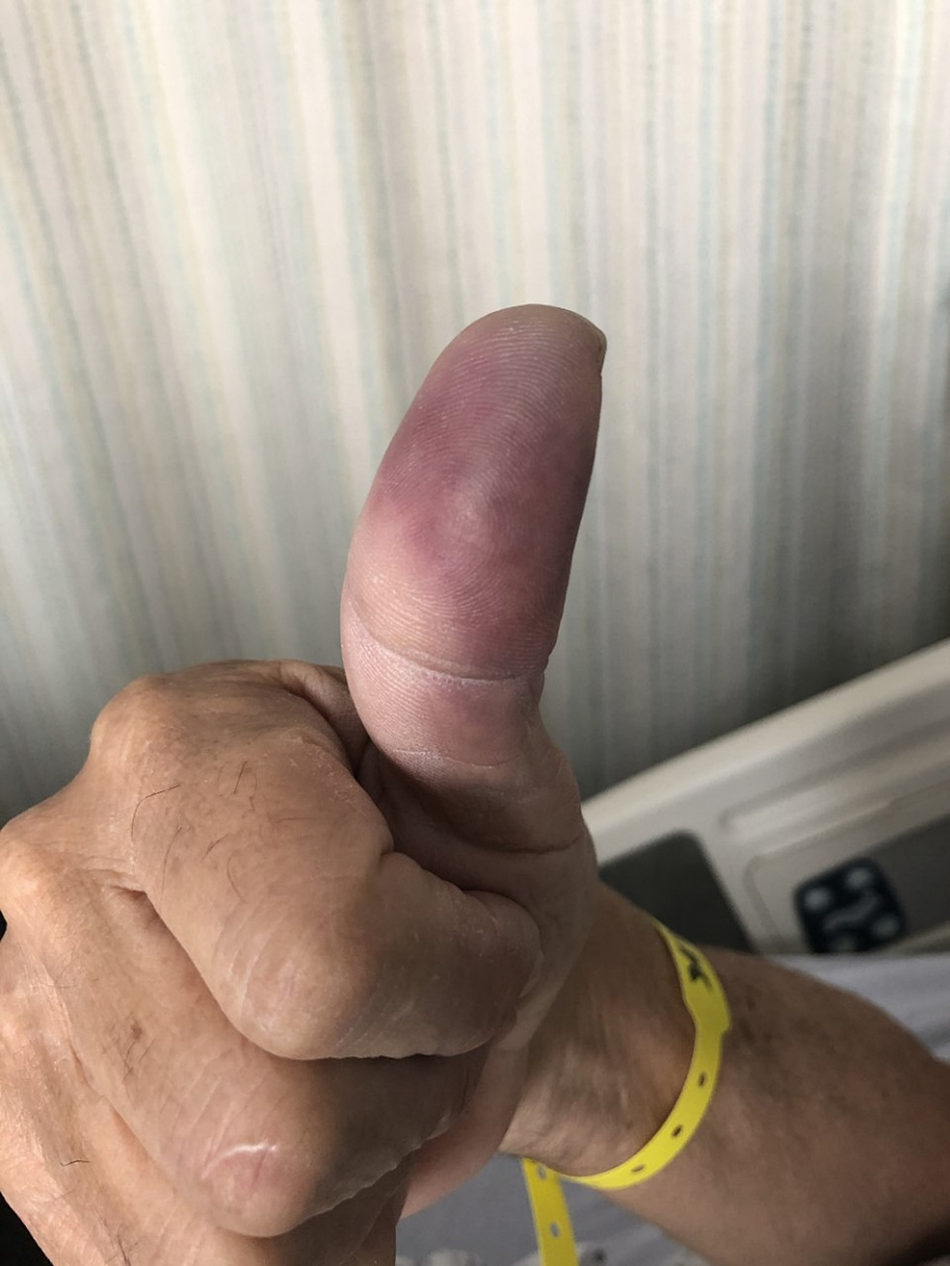
Photograph of the right thumb, showing digital ischemia

A 67-year-old male with a past medical history of type-2 diabetes mellitus and hypertension presented to the ER with dusky bluish discoloration, pain, and coldness on the distal part of the right thumb. The patient had already been seen in the emergency room a week ago due to pain in the distal part of the right thumb. An X-ray of the right hand had been performed at that time, which had shown soft tissue swelling of the right thumb without any evidence of osteomyelitis or fractures. The patient had been diagnosed with felon of the right thumb and discharged on sulfamethoxazole/trimethoprim and ibuprofen. Despite taking these antibiotics, there had been no improvement in his symptoms, and he had noticed worsening pain and discoloration of his thumb. Otherwise, the patient denied any history of trauma. There was no personal or family history of coagulopathy or any rheumatological disorders either. He also denied smoking or using any recreational drugs (cocaine/heroin) or blood thinners. Physical examination showed dusky bluish discoloration of the distal part of the right thumb. It was moderately cold to touch, and there was a clear line of demarcation between the healthy and the ischemic area. Radial, ulnar, and Princeps pollicis pulses were palpable. There were no signs of respiratory distress, and the patient's oxygen saturation was 95-96% on room air. Figure 1 shows a photograph of the right thumb, which shows digital ischemia.

**Figure 2:**
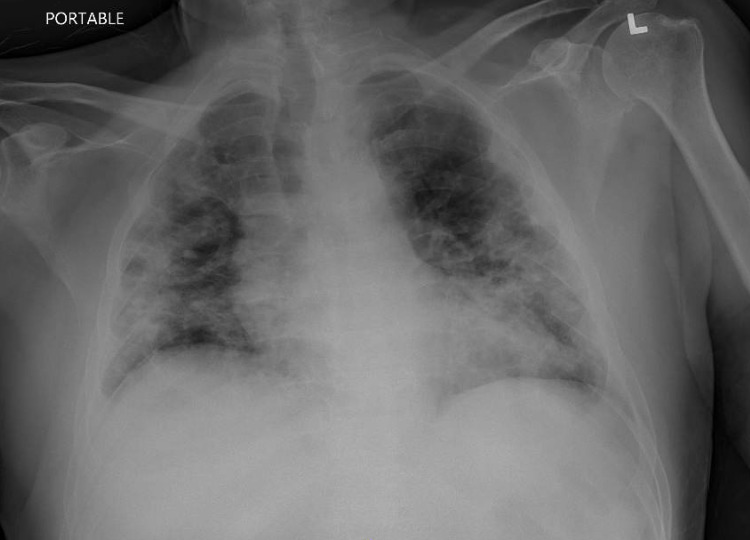
Chest X-ray showing bilateral pneumonia

Laboratory investigations showed elevated levels of inflammatory markers - lactate dehydrogenase (LDH): 241 units/L (normal range: 110-210 units/L); D-dimer: 315 ng/mL (normal range: 0-230 ng/mL); C-reactive protein (CRP): 8 mg/L (normal level: <=5 mg/L); ferritin: 1,471 ng/mL (normal range: 13.0-150.0 ng/mL) (Table 1). Chest X-ray showed bilateral pneumonia (Figure 2). The patient subsequently tested positive for COVID-19.

**Table 1:** Labs showing elevation in the inflammatory markers Hb: hemoglobin; WBC: white blood cells; LDH: lactate dehydrogenase; PT: prothrombin time; APTT: activated partial thromboplastin time

Labs	On admission	On the day of discharge
Hb	15.7 g/dl	13.7 g/dl
WBC	5.5 k/ul	9.0 k/ul
Lymphocytes	0.9 k/ul	0.5 k/ul
D-dimer	315 ng/mL	156 ng/mL
LDH	241 units/L	231 units/L
Ferritin, serum	1,471 ng/mL	1,304 ng/mL
Platelet count	181 k/ul	147 k/ul
PT	14.2 seconds	10.8 seconds
APTT	35.2 seconds	30.6 seconds
INR	1.20	0.92

**Figure 3:**
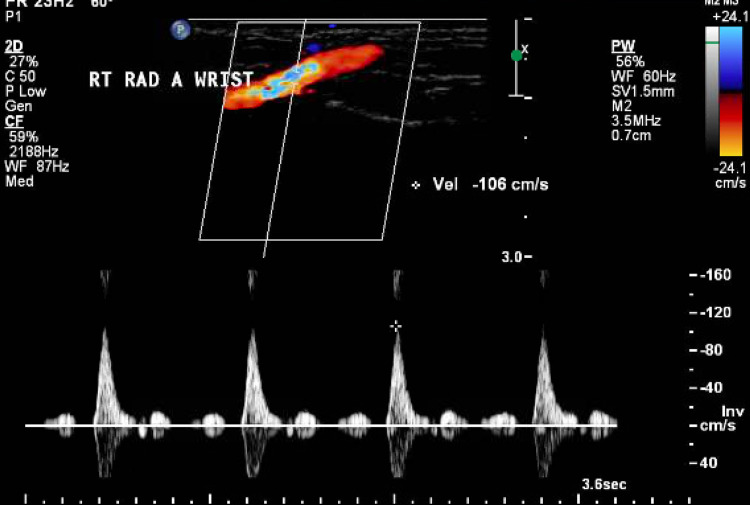
Arterial duplex of the bilateral upper extremities

**Figure 4:**
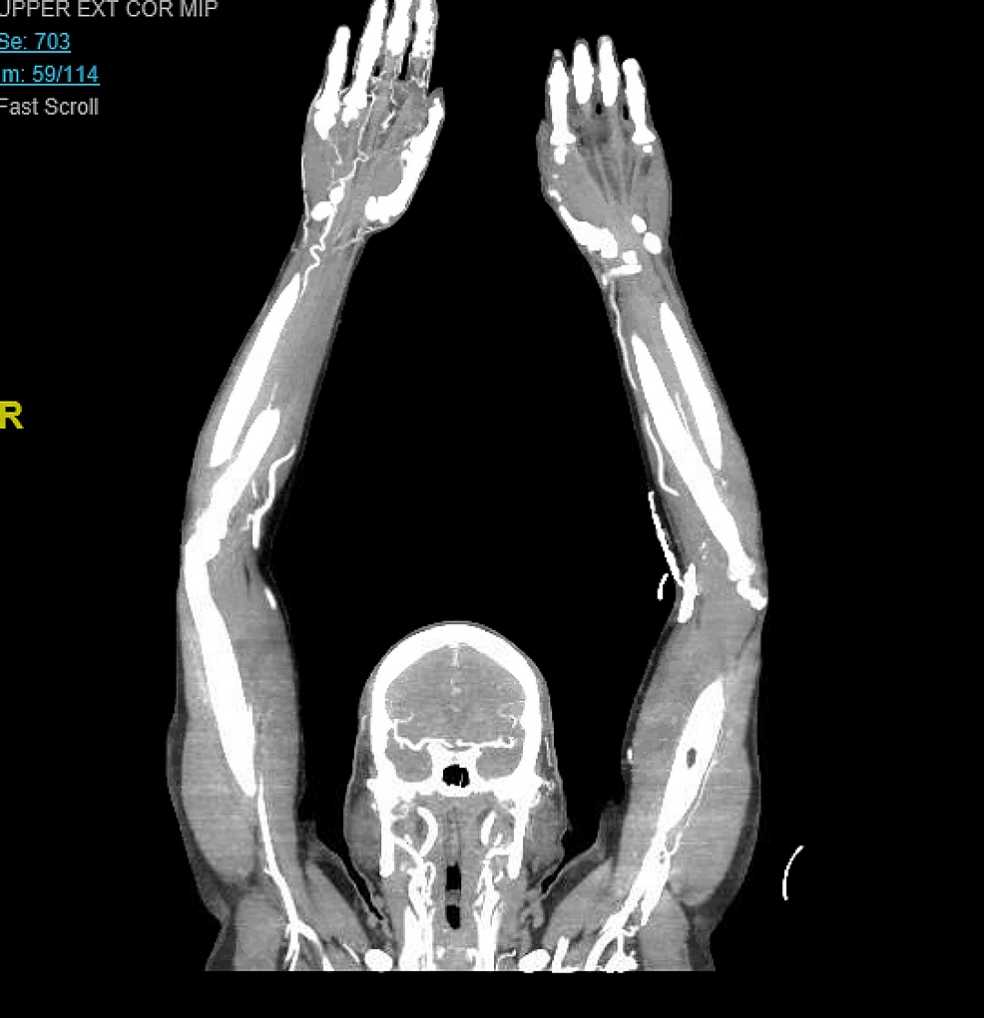
CT angiography of the bilateral upper extremity CT: computed tomography

Vascular surgery was consulted, and the patient was started on a therapeutic dose of enoxaparin and cilostazol. He was also simultaneously treated for COVID-19 with cefepime, azithromycin, Augmentin (amoxicillin/clavulanic acid), dolutegravir, and lamivudine as per hospital protocol. CT angiography of the chest and that of the right upper extremity was performed, which showed no significant abnormality. Over the course of hospitalization, the patient's inflammatory markers trended up, and he required supplemental oxygen via nasal cannula. He received two doses of tocilizumab and one dose of convalescent plasma as well. Figure 3 illustrates the arterial duplex of the bilateral upper extremities, and Figure 4 shows CT angiography of the bilateral upper extremity.

The patient also underwent angiography of the right upper extremity, which showed no arterial occlusion or stenosis. His inflammatory markers gradually trended down. He was later switched from enoxaparin to apixaban. Over the course of his hospital stay, pain and discoloration of his right thumb improved, and he was discharged home on oxygen and apixaban.

The anticardiolipin antibody test was performed, which came back positive for cardiolipin immunoglobulin M (IgM) antibody, which is generally observed in COVID-19-related coagulopathy. The patient had already been discharged when the results came.

## Discussion

Most patients with COVID-19 have a mild to moderate clinical presentation. The severe presentation of the condition predominantly manifests as pulmonary involvement. In Wuhan, China, seven cases of limb ischemia in severely ill patients with COVID-19 pneumonia have been reported. These patients were not in shock and not on any vasopressors, even though they had varying degrees of acral ischemia including plantar plaques and acral bruises [2].

Numerous theories have been proposed regarding ischemia in COVID-19, which include hypoxia-induced microvascular damage and endothelial shedding, and cytokine/inflammation-mediated damage [3]. Endothelial inflammation and dysfunction is the primary cause of injury; additionally, an increased incidence of abnormal coagulation parameters and disseminated intravascular coagulation (DIC) has been noted in patients with COVID-19 [4,5]. All these factors contribute to the risk of thrombosis and ischemic events.

A debate has been ongoing as to whether there is an association between COVID-19 coagulopathy and antiphospholipid (APL) antibodies. A few cases of COVID-19 have been found to have positive APL antibodies, as in our patient. Lung insult is an uncommon phenomenon in APL syndrome, but its catastrophic form [known as catastrophic antiphospholipid syndrome (CAPS)] may cause lung injury and multiorgan failure as well. Still, no clear association has been established between CAPS and COVID-19 coagulopathy [6].

In treating COVID-19-associated coagulopathy, a prophylactic dose of Low-molecular-weight heparin (LMWH) is preferred unless any contraindications such as acute kidney injury are present, in which case unfractionated heparin would be the choice for anticoagulation. LMWH has several anticoagulant properties; for instance, it decreases the release and biological activity of interleukin 6 (IL-6) [7]. LMWH directly binds to SARS-CoV-1, thereby blocking viral replication [8]. It has been reported that COVID-19 patients treated with LMWH have shown lower IL-6 levels and higher lymphocyte counts than those who did not receive LMWH, which strongly indicates the anti-inflammatory property of LMWH [9].

Additionally, a significant reduction in the D-dimer level and fibrinogen degradation products is noted with LMWH use, indicating an improvement in hypercoagulable state in COVID-19 patients [7]. LMWH is beneficial due to its effect of inhibiting the release of IL-6 along with an increase in lymphocyte counts that hinders or blocks the inflammatory cytokine storm. Unfractionated heparin is the treatment of choice for venous thromboembolism in the critical care unit because of its short duration of action and absence of interactions with any of the experimental drugs for COVID-19 [10].

Oral anticoagulants, such as warfarin, dabigatran (direct thrombin inhibitor), apixaban (factor Xa inhibitors), rivaroxaban, edoxaban, and betrixaban, are not used in the treatment of COVID-19-related thrombosis in light of the studies that have shown these drugs' potential interactions with investigational antiviral therapeutics [11].

Though digital/extremity ischemia is routinely seen in ICU/critically ill patients, we discussed an exceptional case of digital ischemia of the right thumb, in a patient with COVID-19 who presented with only digital ischemia without any respiratory symptoms.

## Conclusions

COVID-19 patients can present with various clinical manifestations, with the limb or digital ischemia being one of them. It can be seen in patients without any significant respiratory symptoms. Early diagnosis and treatment with LMWH can improve outcomes given its anticoagulation and anti-inflammatory properties. CT angiography of the chest must be performed in all these patients to rule out pulmonary embolism.
